# Polycystic Ovary Syndrome and Subjective and Objective Assessments of Sleep Quality: A Systematic Review and Meta‐Analysis

**DOI:** 10.1002/hsr2.73223

**Published:** 2026-09-14

**Authors:** Arefeh Tabashiri, Pourya Kanani, Marzieh Saei Ghare Naz, Fahimeh Ramezani Tehrani

**Affiliations:** ^1^ Reproductive Endocrinology Research Center, Research Institute for Endocrine Molecular Biology, Research Institute for Endocrine Sciences Shahid Beheshti University of Medical Sciences Tehran Iran; ^2^ School of Medicine Shahid Beheshti University of Medical Sciences Tehran Iran; ^3^ Foundation for Research & Education Excellence Vestavia Hills Al USA

**Keywords:** meta‐analysis, polycystic ovary syndrome, sleep disturbance, sleep wake disorders

## Abstract

**Background and Aims:**

Sleep disturbances are increasingly recognized in women with polycystic ovary syndrome (PCOS), but the evidence remains inconsistent. We conducted a meta‐analysis to evaluate objective sleep abnormalities and subjective sleep complaints in women with PCOS.

**Methods:**

We systematically reviewed and meta‐analyzed observational studies comparing women with PCOS and women without PCOS. Primary outcomes were objective sleep parameters assessed by polysomnography, while secondary outcomes included subjective sleep quality and daytime sleepiness measured using validated questionnaires, including the Epworth Sleepiness Scale (ESS) and Pittsburgh Sleep Quality Index (PSQI).

**Results:**

Twenty‐one studies involving 1,255 women with PCOS and 1,523 controls were included. Women with PCOS had a significantly higher apnea‐hypopnea index compared to controls (SMD = 1.030, 95% CI: 0.408 to 1.653, *p* = 0.001). Additionally, women with PCOS exhibited significantly higher scores on both PSQI and ESS questionnaires compared to the control group (SMD: 0.435; 95% CI: 0.190 to 0.680, *p* = 0.001, and SMD: 1.263; 95% CI: 0.335 to 2.191, *p* = 0.008, respectively). Meta‐analysis demonstrated longer stage 1 sleep in PCOS group (SMD: 2.356; 95% CI: 0.224 to 4.488, *p* = 0.03). However, REM sleep, stage 2, and stages 3 and 4 showed no significant differences between groups.

**Conclusion:**

This study highlights significant differences in some sleep‐disordered breathing indices, sleep structure, and objective sleep quality measures between women with PCOS and control groups. Subjective sleep measures demonstrated more pronounced differences than some objective assessments, suggesting a greater impact on perceived sleep disturbances.

## 1 Introduction

Polycystic ovary syndrome (PCOS) is a significant public health concern, affecting 5%–18% of women of reproductive age [1]. It is estimated that up to 70% of the cases remain undiagnosed [2]. PCOS is the most common cause of anovulation and infertility worldwide [3]. It is characterized by hyperandrogenism, ovulatory dysfunction, and polycystic ovarian morphology, as outlined in the 2023 version of international evidence‐based guideline for the management of PCOS [4]. Beyond its reproductive manifestations, PCOS is associated with metabolic and psychological complications that collectively impair quality of life [5].

Among PCOS complications, sleep disturbances have gained increasing attention lately [6] and improving sleep quality is a part of modification of lifestyle aspects in the management of PCOS. Improved sleep quality can mitigate hormonal imbalance, insulin resistance, inflammation, and weight gain [7, 8, 9, 10]. Additionally, it is associated with enhanced mental and reproductive health [11, 12]. Despite the role of sleep quality on PCOS symptoms and complications, PCOS itself is associated with sleep disturbances [13]. Sleep disturbances in women with PCOS present in various forms. Changes in sleep quality, structure, and duration, as well as sleep‐related disorders, including obstructive sleep apnea (OSA) and sleep‐disordered breathing (SDB), are reported to be more common in women with PCOS [13, 14]. The underlying pathophysiology is multifactorial. Hormonal imbalance, metabolic dysregulation, psychological stress, elevated androgen levels, insulin resistance, and the chronic nature of PCOS are considered to contribute to the development of sleep disorders [14].

A recent meta‐analysis assessed the risk of OSA in women with PCOS. The study revealed a higher risk of OSA and more severe symptoms in PCOS population compared to the control group [15]. These findings were in consistent with previous systematic review and meta‐analysis studies [16, 17]. Although these studies provided robust evidence on sleep disorders (mainly OSA) in women with PCOS, they failed to systematically review both subjective and objective sleep quality in this population. Therefore, the broader spectrum of sleep disturbance remained poorly understood.

Although previous studies reported sleep disturbances among women with PCOS [18], types of common and uncommon sleep disorders among them remain incompletely characterized. Furthermore, existing literature is inconsistent: while several studies indicate a high prevalence of sleep disorders such as OSA and insomnia among PCOS, others suggest that these associations may be overestimated, potentially due to selection bias toward severely affected women who referred to tertiary care centers rather than the broader PCOS population [17, 19, 20].

To extend previous reviews, we conducted a systematic review and meta‐analysis to synthesize existing research to provide a clearer picture of both objective sleep disturbances and subjective sleep complaints in this population. Such a synthesis is essential for developing targeted interventions and improving clinical management strategies to enhance the well‐being of women with PCOS.

## 2 Materials and Methods

### 2.1 Study Design

This systematic review and meta‐analysis was conducted in accordance with the Preferred Reporting Items for Systematic Reviews and Meta‐Analyses (PRISMA) guidelines [21].

### 2.2 Search Strategy

A comprehensive literature search was performed in PubMed/MEDLINE, Scopus, and Web of Science databases from inception to 20 August 2024. Databases were searched using combinations of MeSH terms related to PCOS and sleep. The full search strategy is detailed in Supplementary File 1.

### 2.3 Inclusion and Exclusion Criteria

Inclusion criteria included:

1. Studies on women diagnosed with PCOS according to Rotterdam criteria [22], the criteria of the National Institutes of Health (NIH) [23], or other valid and reliable criteria.
2. Studies assessing prevalence, incidence, and risk factors of sleep disturbances in women with PCOS.
3. Observational studies (cross‐sectional, cohort, and case‐control studies) and clinical trials.

Exclusion criteria included:

1. Studies not involving human subjects.
2. Studies with insufficient data on PCOS or sleep disturbances.
3. Articles not available in the English language.
4. Articles with no available full‐text.
5. Studies not published in peer‐reviewed journals (the peer‐review status of journals was verified through indexing in recognized databases).
6. Studies focusing on populations with comorbid conditions that could independently affect sleep, such as severe psychiatric disorders or other endocrine disorders.
7. Case reports, reviews, editorials, and commentaries.

### 2.4 Outcomes

The primary outcomes were the polysomnography measures (objective measures) as outlined by the American Academy of Sleep Medicine [24], including:

1. Total sleep time (min)
2. Sleep latency
3. Rapid eye movement (REM) sleep latency
4. Wake after sleep onset
5. Sleep architecture (the percentage of total sleep time in REM sleep, stage 1 sleep, stage 2 sleep, and slow wave sleep (stage 3 and 4 combined).

The secondary outcome is subjective sleep measures, as assessed by standard sleep questionnaires, in women with PCOS compared with controls.

### 2.5 Study Selection

All retrieved references were imported into EndNote (version X9, Clarivate Analytics) for management. Duplicate records were identified and removed using EndNote's automated function and manual checking. Two independent reviewers screened titles and abstracts of the articles. After removing the replicated publications, studies were excluded from further assessment if their titles or abstracts clearly indicated irrelevance. Full‐text articles of potentially relevant articles were assessed for inclusion. Any disagreements were resolved by discussion or consultation with a third reviewer.

### 2.6 Data Extraction

The following relevant data were extracted and imported into an Excel datasheet:

1. Study characteristics, including title, first author, country, year of publication, and study design.
2. Demographic data, including sample size and age.
3. Sleep‐related questionnaires, scores, and indices, including Pittsburgh Sleep Quality Index (PSQI) score, Berlin Questionnaire (BQ) score, Epworth Sleepiness Scale (ESS) score, apnea index, hypopnea index, apnea‐hypopnea index, arousal index, Athens Insomnia Scale (AIS), respiratory disturbance index (RDI), SDB, periodic limb movements (PLM), etc.
4. Sleep‐related parameters, including sleep duration and quality, sleep efficiency, sleep structure disturbances, daytime dysfunction, sleep induction time, sleep architecture (REM and non‐REM duration), etc.

### 2.7 Quality Assessment

Two reviewers (AT and PK) independently evaluated the quality of included studies using the Newcastle‐Ottawa Scale (NOS) [25] for observational studies. Scores were recorded and managed in Microsoft Excel. Any disagreement was resolved through discussion or, if necessary, consultation with a third reviewer (MS). NOS consists of three distinct sections: selection, comparability, and outcome assessment. The first section evaluates the adequacy of the study group's selection, with scores ranging from 0 to 4 stars. The second section assesses the degree to which confounding variables have been controlled and assesses comparability of the study groups, with the score ranging from 0 to 2 stars. The final section evaluates either the outcome (in cohort and cross‐sectional studies) or the exposure (in case‐control studies), with scores ranging from 0 to 3 stars. The total score across all three sections varies from 0 to 9. The interpretation of the score is commonly 7–9 stars for high‐quality studies, 4–6 stars for moderate studies, and 0–3 stars for low‐quality studies [25].

### 2.8 Certainty of Evidence

The certainty of the evidence for all objective and subjective outcomes was assessed using the Grading of Recommendations, Assessment, Development and Evaluations (GRADE) approach. This process was conducted by two independent reviewers (AT and PK), and consensus was achieved through discussion.

Since all of the included studies were observational (cross‐sectional, case‐control, cohort), the initial certainty of evidence for all outcomes was set at low. The certainty was then evaluated and downgraded across five domains:

1. Risk of Bias (RoB) was assessed based on the quality rating derived from the NOS. Evidence was downgraded one level for serious flaws and two levels for very serious flaws.
2. Inconsistency (Heterogeneity) was evaluated based on I^2^ values. We applied one level of downgrade for I^2^ > 50% and two levels for I^2^ > 90%.
3. Indirectness was assessed to determine if the study population, intervention, or outcome differs from the question posed by the review.
4. Imprecision was evaluated based on 95% CI. The certainty was downgraded by one level if the 95% CI crossed the null effect or if the CI was too wide to be clinically conclusive.
5. Publication Bias was assessed using visual inspection of funnel plots and statistical tests (Egger's and Begg's tests). We applied downgrading by one level if Egger's test indicated significant bias (*p *< 0.05).

### 2.9 Statistics

All analyses adhered to PRISMA guidelines for meta‐analytic reporting. Pooled effect sizes were estimated using a fixed‐effects approach when heterogeneity was low and using a random‐effects model when heterogeneity was moderate to high. Effect estimates are presented as standardized mean differences (SMDs) accompanied by 95% confidence intervals (CIs). Heterogeneity across studies was quantified using the I^2^ statistic, with thresholds of 25%, 50%, and 75% denoting low, moderate, and high levels, respectively. Furthermore, heterogeneity was tested using Cochran's Q statistic, where a two‐sided *p*‐value < 0.10 was taken as evidence of significant heterogeneity. To assess the stability of the results, sensitivity analyses were performed by removing studies judged to carry a high risk of bias and/or identified as outliers. Potential publication bias was evaluated through visual inspection of funnel plots along with Egger's and Begg's tests, with a two‐sided *p*‐value < 0.05 flagged as suggestive of bias. For outcomes with a limited number of studies (*n* < 10), the power of Egger's and Begg's tests to detect publication bias is insufficient. So, these results must be interpreted with caution. Statistical significance for all tests was defined as a two‐sided *p*‐value < 0.05. Analyses were performed using MedCalc statistical software, version 23.2.1 (MedCalc Software Ltd, Ostend, Belgium).

## 3 Results

### 3.1 Literature Selection

**Figure 1:**
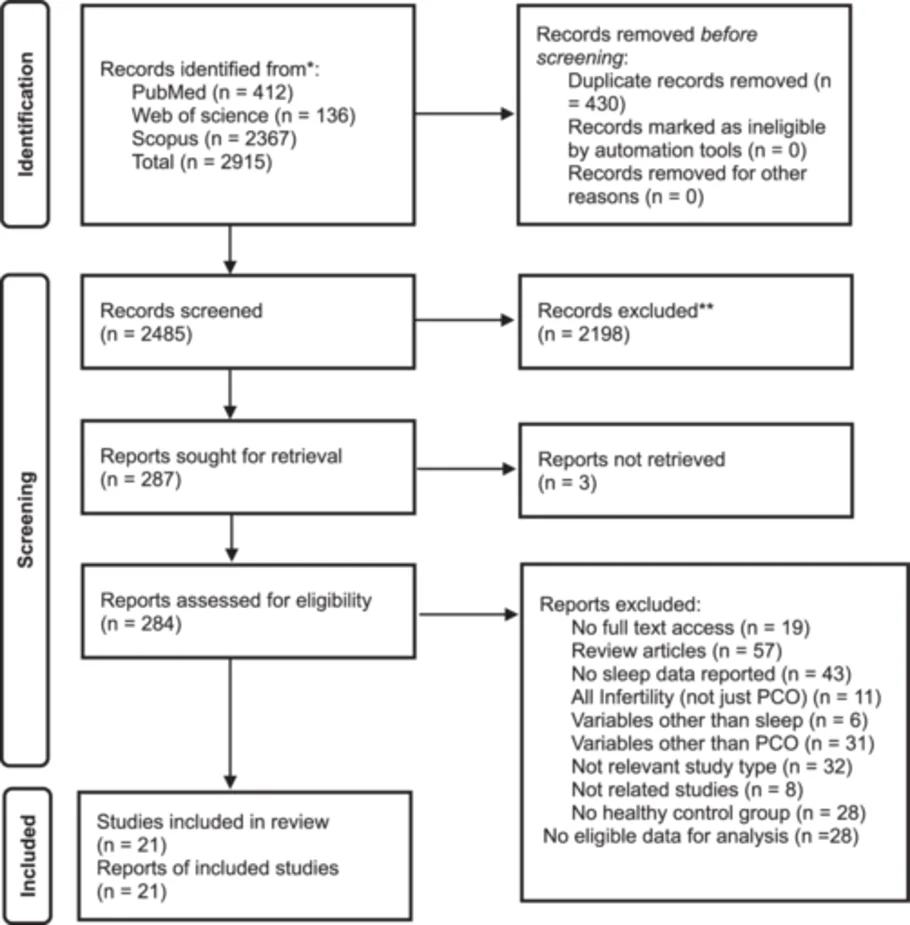
PRISMA flow diagram illustrating the process of study selection for the systematic review and meta‐analysis.

Following a systematic search through PubMed/MEDLINE, Scopus, and Web of Science, a total of 2,915 records were identified. After removing duplicates, 2,485 records remained for the initial screening. After a thorough screening of titles and abstracts, a total of 2,198 studies were excluded. Of the remaining 287 records, 266 were excluded through full‐text screening due to lack of full‐text access, article type, lack of quantitative data on primary and secondary outcomes, and failure to report the required variables. Ultimately, 21 studies were included, 12 of which were cross‐sectional [26, 27, 28, 29, 30, 31, 32, 33, 34, 35, 36, 37]. Seven case‐control studies [38, 39, 40, 41, 42], and two cohorts [43, 44] were included in this review and meta‐analysis. Detailed information on the study selection process is available in the PRISMA flow diagram (Figure 1).

### 3.2 Study Characteristics

The baseline characteristics of the included studies are extracted and are available in Table 1. Of the 21 studies, seven studies [26, 27, 28, 29, 30, 31, 43] included adolescents, and 14 included adults [32, 33, 34, 37, 38, 39, 40, 41, 42, 44, 45]. Among the included studies, 11 studies focused on overweight or obese women [26, 27, 28, 29, 30, 31, 32, 39, 43, 45, 46], of which in three studies obese cases were compared to normal‐weight controls [30, 31, 32]. Three studies examined normal‐weight women [36, 38, 41]. Six studies included both normal‐weight and overweight or obese women [33, 34, 35, 37, 40, 42]. One study did not report BMI of the included groups [44]. All studies reported the BMI in metric units. One study reported BMI percentile [44]. Regarding subjective sleep quality, six studies employed only the PSQI [34, 37, 38, 40, 41, 44], one of which utilized PSQI in combination with an alternative questionnaire [34]. Three utilized only the ESS [31, 36, 39], and one study used both of the ESS and PSQI [42]. Two studies used three questionnaires of PSQI, ESS, and BQ [32, 33], while one used both ESS and BQ [35]. Six studies didn't assess subjective sleep quality [26, 27, 28, 29, 30, 43], and two studies employed alternative assessments [45, 46]. In terms of PCOS diagnostic criteria both the Rotterdam and National Institutes of Health (NIH) criteria were utilized, five studies followed the NIH criteria [26, 27, 28, 29, 30, 45, 46], 12 studies adhered to the Rotterdam criteria [31, 32, 33, 34, 35, 36, 37, 40, 41, 42, 43, 44], and four studies used criteria other than the two prior ones [38, 39, 45, 46].

**Table 1:** Study characteristics of the included studies.

Author	Country and study period	Study design	PCOS criteria	Control definition	Population number age (mean, years)	Exclusion criteria	BMI (kg/m^2^)	Sleep questionnaire
Azizi et al. (1)	Iran 2020	Cross sectional	Rotterdam criteria	Healthy women with male infertility	Case: No.: 201 Age: 27.86 ± 5.84 Control No.: 199 Age: 28.06 ± 6.51	Non‐PCOS, ages of < 15 or > 40, non‐married, presence of non‐classic adrenal hyperplasia, thyroid dysfunction, Hyperprolactinemia, smoker, problems in speaking or listening, non‐Iranian, taking any prescription medication (except allergy medications and occasional pain medications) for at least 3 months before entering the study	PCOS: 22.73 ± 9.62 Controls: 23.95 ± 4.96	PSQI
Kangwolkij et al. (2)	Thailand 2021	Cross sectional	Rotterdam criteria	Women without PCOS (1) having regular menstruation with menstrual duration between from 21 to 35 days, (2) no evidence of clinical or biochemical hyperandrogenism, and (3) no underlying disease associated with sleep disturbance	Case: No.: 94 Age: 27.00 (no SD was reported) Controls: No.: of 47 Age: 27.00 (no SD was reported)	Women with other conditions related to sleep disorder including smoking, alcohol or caffeine drinking, sedative drug use, and receiving any medication with a side effect of drowsiness. Receiving hormonal contraceptive 3 months prior to study	PCOS: 26.1 ± 2.57 Control: 25.1 ± 2.07	BQ, ESS, PSQI
De Sousa et al. (3)	Germany 2009	Cross sectional	NIH criteria	Patients with ruled out sleep‐related breathing disorders and normal menstrual cycles (28–35 days) and no clinical signs of androgen excess	Case: No.: 22 Age: 5.2 ± 1.3 Control No.: 11 Age: 15 ± 1	Nonclassical adrenal 21‐hydroxylase deficiency, androgen‐secreting tumors, and Cushing's syndrome were excluded by appropriate tests before the diagnosis of PCOS was made.	PCOS: 31.7 ± 6.2 Controls: 34.8 ± 8.7	Not assessed
De Sousa et al. (4)	Germany 2011	Cross sectional	NIH criteria	Patients had normal menstrual cycles (28–35 days) and no clinical signs of androgen excess, thereby excluding PCOS by the definition of the NIH. All controls were without evidence of other diseases and were currently not taking any medications	Case: No.: 15 Age: 15.30 ± 1.20 Control: No.: 16 Age: 19.40 ± 0.70	Women with conditions such as non‐classical adrenal 21‐hydroxylase deficiency, androgen‐secreting tumors, and Cushing's syndrome were excluded by appropriate tests before the diagnosis of PCOS was made	PCOS: 32.90 ± 6.40 Controls: 21.70 ± 2.30	Not assessed
De Sousa et al. (5)	Germany 2010	Cross sectional	NIH criteria	Outpatients aged 13–17; healthy and with no history pointing towards sleep‐related breathing disorders and with normal menstrual cycles (28–35 days) and no clinical signs of androgen excess	Case: No.: 31 Age: 15.00 ± 1.00 Control: No.: 19 Age: 15.20 ± 1.10	Conditions such as non‐classical adrenal 21‐hydroxylase deficiency, androgen‐secreting tumors and Cushing's syndrome were excluded by appropriate tests before the diagnosis of PCOS was made	PCOS: 32.70 ± 6.20 Controls: 32.40 ± 4.00	Not assessed
De Sousa et al. (6)	Germany 2011	Cross sectional	NIH criteria	Healthy obese adolescents without PCOS or metabolic syndrome aged 13–17 years	Case: No.: 14 Age: 15.70 ± 1.90 Control: No.: 19 Age: 15.30 ± 1.00	Conditions such as nonclassical adrenal 21‐hydroxylase deficiency, androgen‐secreting tumors, and Cushing syndrome were excluded by appropriate tests	PCOS: 36.20 ± 6.20 Controls: 34.40 ± 6.50	Not assessed
De Sousa et al. (7)	Germany 2012	Cross sectional	NIH criteria	Healthy obese adolescents without PCOS aged 13 to 17 years	Case: No.: 35 Age: 15.16 ± 1.04 Control: No.: 19 Age: 15.23 ± 1.09	Conditions such as nonclassical adrenal 21‐hydroxylase deficiency, androgen‐secreting tumors, and Cushing syndrome were excluded by appropriate tests	PCOS: 33.22 ± 5.88 Controls: 32.36 ± 3.95	Not assessed
El‐Sharkawy et al. (8)	Egypt 2014	Cross sectional	Rotterdam criteria	Untreated group	Case: No.: 30 Age: 15.90 ± 0.36 Control No.: 30 Age: 15.70 ± 0.12	Patients with any chronic diseases such as hypertensive and hepatic patients and those with malignancy or congenial adrenal hyperplasia	PCOS: 35.25 ± 0.73 Control: 21.47 ± 0.55	Sleep disturbances scaleESS
Hachul et al. (9)	Brazil 2018	Cross sectional	Rotterdam criteria	Regular menstrual cycle of 28–30 days, normal BMI and in the follicular phase of the menstrual cycle	Case: No.: 30 Age: 29.70 ± 1.20 Control: No.: 14 Age: 27.90 ± 1.70	neurologic conditions and/or being under psychiatric treatment; use of medication for chronic diseases that might interfere with the study results; participation in another clinical study or having participated in a clinical study within a period of 3 months; being a carrier of a disease; having a history of stroke; use of hypnotic, psychotropic, psychostimulant, and/or analgesic drugs; use of hormonal contraceptives; and presence of dysmenorrhea or endometriosis that may interfere with sleep patterns	PCOS: 34.30 ± 1.10 Control: 22.40 ± 1.60	PSQI, BQ, ESS
Karasu et al. (10)	Turkey 2021	Cross sectional	Rotterdam criteria	Healthy controls	Case: No.: 111 Age: 25.13 ± 5.82 Control: No.: 108 Age: 26.40 ± 0.40	Parous women and night shift workers	PCOS: 26.47 ± 5.10 Control: 26.50 ± 5.00	PSQI, Morningness Eveningness Questionnaire
Suri et al.(11)	India 2015	Cross sectional	Rotterdam criteria	Age‐matched women who attended the gynecology outpatient department with other complaints such as vaginal discharge, dysuria, and pelvic organ prolapse were recruited. All of the control group experienced regular menstrual cycles and did not meet the standard diagnostic criteria for PCOS	Case: No.: 50 Age: 27.90 ± 6.44 Control: No.: 100 Age: 27.90 ± 6.05	Women taking any form of treatment for PCOS were not included in the study. Patients with thyroid disorders, hyperprolactinemia, and congenital adrenal hyperplasia, with history of smoking, and with neurological or psychiatric disorders were also excluded from the study.	PCOS: 28.00 ± 4.01 Control: 25.30 ± 2.93	BQ, ESS
Yang et al. (12)	Taiwan 2008	Cross sectional	Rotterdam criteria	Age‐matched and BMI‐matched women who did not have PCOS were recruited as a control group from the same community during the same period. Women were excluded from the control group if they had irregular menstruation or oligomenorrhea, abnormal serum thyroid‐stimulating hormone or prolactin, or biochemical hyperandrogenemia	Case: No.: 18 Age: 29.10 ± 1.43 Control: No.: 10 Age: 31.60 ± 3.87	Women taking any medication affecting the hypothalamic–pituitary–ovarian axis within the last 6 months, women who had been pregnant within the last year, and women with diabetes, hypertension, other diseases associated with obesity, hyperprolactinemia, abnormal thyroid function tests, and congenital adrenal hyperplasia	PCOS: 21.70 ± 0.57 Control: 20.90 ± 0.58	ESS
Li et al. (13)	China 2022	Case‐Control	Rotterdam criteria	Women who were infertile due to tubes or male factors with normal levels of sex hormones and AMH. All women had not taken oral contraceptives for at least three months.	Case: No.: 35 Age: 26.93 ± 1 Control: No.: 36 Age: 28.06 ± 1	Congenital uterine abnormalities, gynecological tumors and other endocrine diseases, such as hyperthyroidism, hypothyroidism, hyperprolactinemia, Cushing's syndrome, etc.	PCOS: 22.33 ± 0.15 Control: 21.13 ± 0.08	PSQI
Eyupoglu et al. (14)	Turkey 2020	Case‐Control	PCOS Health‐Related Quality of Life Questionnaire (PCOSQ)	Women with regular menstrual cycles and no clinical signs of hyperandrogenism or other chronic disease	Case: No.: 232 Age: 23.00 Control: No.: 157 Age: 23.00	Women under 18 or over 45 years of age, and/or currently pregnant, breastfeeding, or menopausal	PCOS: 21.90 ± 2.60 Control: 21.50 ± 2.90	PSQI
Fogel et al. (15)	USA 2000	Case‐Control	Chronic oligomenorrhea (six or fewer menses per year) along with elevated serum androgen levels (total or biologically available testosterone levels) non‐classical 21‐hydroxylase deficiency was excluded by a 1‐h ACTH stimulation test	18 age‐ and weight‐matched controls, not taking medications, normal menstrual cycles (28–35 days), no clinical signs of androgen excess, and normal serum levels of androgens.	Case: No.: 18 Age: 31.10 ± 1.30 Control: No.: 18 Age: 32.30 ± 1.30	—	PCOS: 36.90 ± 1.30 Control:36.90 ± 1.40	ESS
Li et al. (16)	China 2020	Case‐Control	Rotterdam criteria	Healthy female controls matched by age and education. All controls were 18 to 35 years old and had regular menstrual cycles (28–30 days). Controls with a personal history of diabetes or hypertension and a family history of PCOS were excluded.	Case: No.: 41 Age: 25.29 ± 3.15 Control: No.: 41 Age: 26.22 ± 2.59	(1) current or recent (last six months) hormonal treatment; (2) pregnant or breastfeeding; (3) other diseases that may cause hyperandrogenism or anovulation, such as congenital adrenal hyperplasia, androgen secreting tumor, prolactinoma, Cushing's syndrome and thyroid disease; (4) history of craniocerebral injury, mental disorders, chronic diseases such as diabetes, hypertension, rheumatoid arthritis, cancer or other factors that may affect cognition; and (5) contraindications to MRI examinations such as a pacemaker, internal metal and coronary artery stenting	PCOS: 24.62 ± 4.88 Control: 20.31 ± 1.81	PSQI
Shreeve et al. (17)	United Kingdom 2013	Case‐Control	Rotterdam criteria	Females aged between 18 and 40 years with regular menstrual cycles and no history of subfertility and psychiatric disorder	Case: No.: 26 Age: 29.80 ± 3.70 Control: No.: 26 Age: 26.30 ± 5.60	Night shift workers and pregnant women were excluded from the study, as were women who have additional related pathologies, such as malignancy, thyroid disease, hyperprolactinemia or adrenal gland disease	PCOS: 29.30 ± 8.20 Control: 24.60 ± 3.30	PSQI, ESS
Vgontzas et al. (18)	USA 2001	Case‐Control	Presence of chronic anovulation (six or fewer menstrual periods per year) inassociation with elevated circulating androgen levels	Premenopausal women (age range, 20–42 years; BMI range, 16.1–59.9) selected from a general randomized sample	Case: No.: 53 Age: 30.40 ± 0.90 Control: No.: 452 Age: 32.10 ± 0.30	appropriate tests (?)	PCOS: 38.70 ± 1.10 Control: 26.40 ± 0.30	Sleep Disorders Clinic criteria made by a Sleep Disorders Medicine specialist
Vgontzas et al. (19)	USA 2006	Case‐Control	Women with presence of chronic anovulation (6 or fewer menstrual periods per year) in association with elevated circulating androgen levels (total testosterone more than 201.1 nmol/L and/or free and weakly bound testosterone more than 55.5 nmol/L)	Obese and normal‐weight controls	Case: No.: 42 Age: 29.60 ± 0.90 Control: No.: 17 Age: 35.70 ± 1.00		PCOS: 38.70 ± 1.40 Control: 36.90 ± 1.00	Subjective criteria
Nandalike (20)	USA 2012	Cohort	Rotterdam criteria	Age and BMI Z‐score matched females without PCOS and matched males who underwent polysomnography during the same time period were identified through the sleep disorders center database	Case: No.: 28 Age: 16.80 ± 1.90 Control: No.: 28 Age: 17.10 ± 1.80	Women with other conditions that could mimic PCOS such as: Cushing's syndrome, late onset adrenal hyperplasia, or androgen producing neoplasm, were excluded.	PCOS: 44.80 ± 8.80 Control: 40.20 ± 4.70	Not assessed
Su et al. (21)	USA 2017	Cohort	Rotterdam criteria	Females aged between 23 and 40 years with regular menstrual cycles and no history of subfertility	Case: No.: 129 Age: 29.03 ± 3.26 Control: No.: 156 Age: 31.72 ± 3.86	Women with additional pathologies related to high androgen, such as congenital adrenal hyperplasia, Cushing syndrome malignancy; testosterone‐producing tumors	Not measured	PSQI

The countries of origin of the studies are available in Table 1. Six studies were done in Europe [26, 27, 28, 29, 30, 42], five in North America [39, 43, 44, 45, 46], five in Asia [33, 35, 36, 40, 41], and four in the Middle East [31, 34, 37, 38]. Additionally, one study was included from Central and South America [32]. Inclusion and exclusion criteria of the studies are also summarized in Table 1.

Extracted data from included studies are available in Table S1 in the Supplementary Table 1 [see Supplementary file 2].

### 3.3 Quality Assessment

The total score of the cross‐sectional studies ranged from 6 to 9. Of the 21 studies evaluated, nine were rated moderate, and the remaining 12 were rated as high‐quality. The quality assessment score for the two cohort studies was 6, indicating satisfactory quality. More details on the quality assessment are available in Table 2.

**Table 2:** Quality assessment of the included studies.

Study	Total scale	Selection	Comparability	Outcome
Azizi et al. [37]	High	★★★★	★★	★★
Kangwolkij et al. [33]	High	★★★★	★★	★★
De Sousa et al. [26]	Moderate	★★	★	★★★
De Sousa et al. [30]	Moderate	★★	★	★★★
De Sousa et al. [28]	Moderate	★★	★	★★★
De Sousa et al. [29]	Moderate	★★	★	★★★
De Sousa et al. [27]	Moderate	★★	★	★★★
El‐Sharkawy et al. [31]	Moderate	★★	★	★★★
Hachul et al. [32]	High	★★★	★★	★★
Karasu et al. [34]	Moderate	★★	★	★★★
Suri et al.[35]	High	★★★	★	★★★
Yang et al. [36]	High	★★★★	★★	★★★
Li et al. [41]	High	★★★★	★★	★
Eyupoglu et al. [38]	High	★★★★	★	★★
Fogel et al. [39]	High	★★★★★	★★	★★
Li et al. [40]	High	★★★★	★★	★★
Shreeve et al. [42]	High	★★★★	★★	★★
Vgontzas et al. [45]	High	★★★	★★	★★
Vgontzas et al. [46]	High	★★★	★★	★★
Nandalike [43]	Moderate	★★★	★★	★
Su et al. [44]	Moderate	★★★	★	★★

### 3.4 Primary Outcomes: Objective Measures

#### 3.4.1 Apnea Index

**Figure 2:**
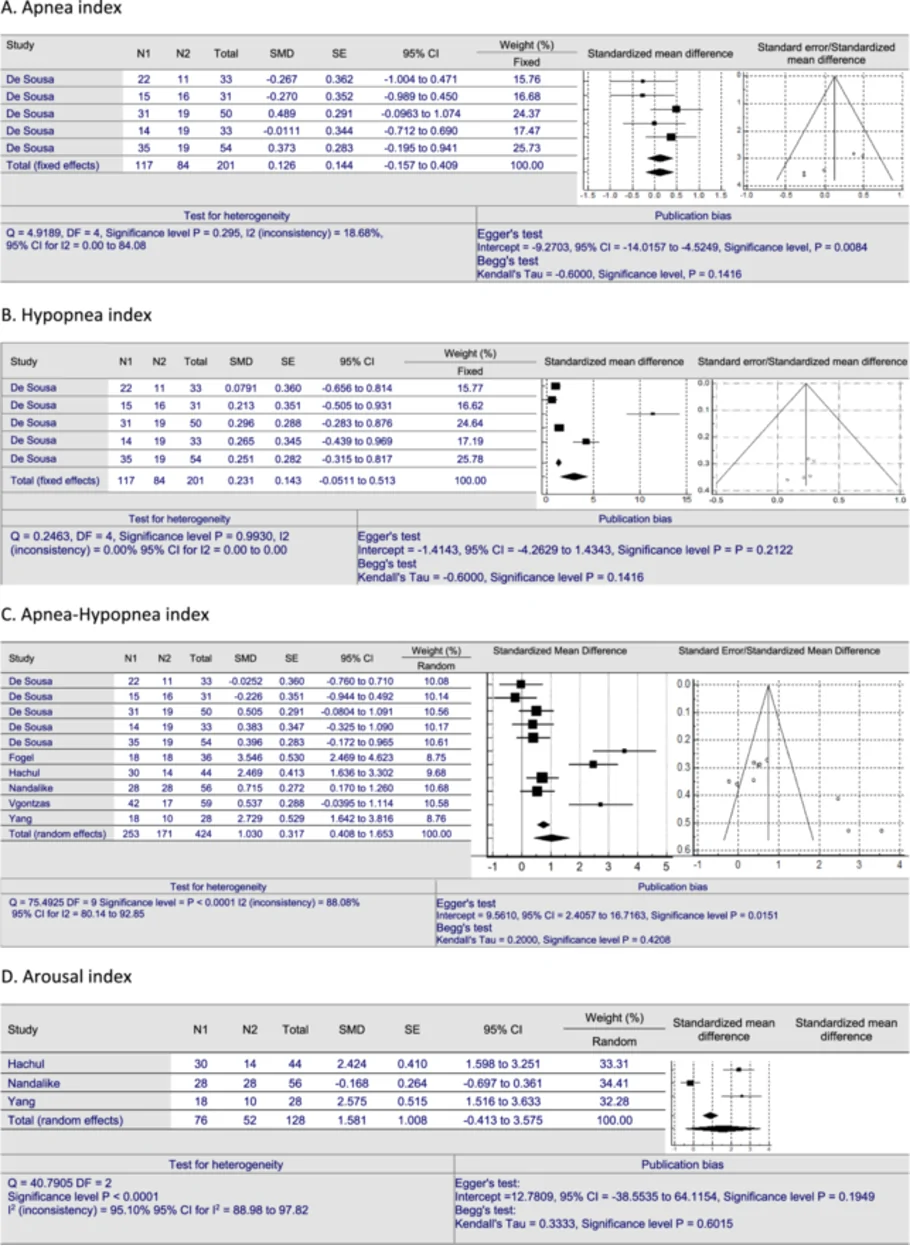
Forest plots of objective polysomnographic sleep‐disordered breathing measures comparing women with polycystic ovary syndrome (PCOS) and controls. Effect sizes are presented as standardized mean differences (SMDs) with 95% confidence intervals (CIs). Funnel plots show the assessment of publication bias. Panels represent the apnea index (A), hypopnea index (B), apnea‐hypopnea index (C), and arousal index (D).

The meta‐analysis included five studies, with a total of 201 participants (117 in the PCOS group and 84 in the control group). The overall effect size for the fixed effects model was 0.126 (95% CI: −0.157 to 0.409, *p *= 0.38), indicating no significant difference between the two groups (Figure 2). The heterogeneity analysis showed no significant heterogeneity among the included studies (I^2^ = 18.68%, *p *= 0.30). The publication bias tests indicated potential bias (Egger's test *p *= 0.008, Begg's test *p *= 0.14) (Figure 2).

#### 3.4.2 Hypopnea Index

The analysis included five studies. The analysis of hypopnea index in PCOS patients compared to the control group revealed non‐significant findings. The overall effect size for the fixed effects model was 0.231 (95% CI: −0.0511 to 0.513, *p *= 0.11), indicating no significant difference between the two groups (Figure 2). The heterogeneity analysis showed no significant heterogeneity among the included studies (I^2^ = 0.00%, *p *> 0.99). The publication bias tests (Egger's and Begg's) did not show significant bias (Figure 2).

#### 3.4.3 Apnea‐Hypopnea Index

The meta‐analysis included ten studies, with a total of 424 participants (253 in the PCOS group and 171 in the control group). The analysis of the apnea‐hypopnea index in PCOS patients compared to the control group revealed significant findings, supporting the presence of a significant difference between the two groups (SMD = 1.030, 95% CI: 0.408 to 1.653, *p *= 0.001) (Figure 2). The heterogeneity analysis showed high heterogeneity among the included studies (I^2^ = 88.08%, *p *< 0.001). The publication bias tests indicated potential bias (Egger's test *p *= 0.015, Begg's test *p *= 0.42) (Figure 2).

#### 3.4.4 Arousal Index

Analysis of the arousal index in PCOS patients compared with the control group showed no significant differences. The random effects model yielded a larger effect size of 1.581 (95% CI: −0.413 to 3.575, *p *= 0.12), which was not statistically significant. (Figure 2). The heterogeneity analysis indicated high heterogeneity among the included studies (I^2^ = 95.10%, *p *< 0.001). The publication bias tests (Egger's and Begg's) did not show significant bias. (Figure 2).

### 3.5 Sleep Architecture

#### 3.5.1 REM

**Figure 3:**
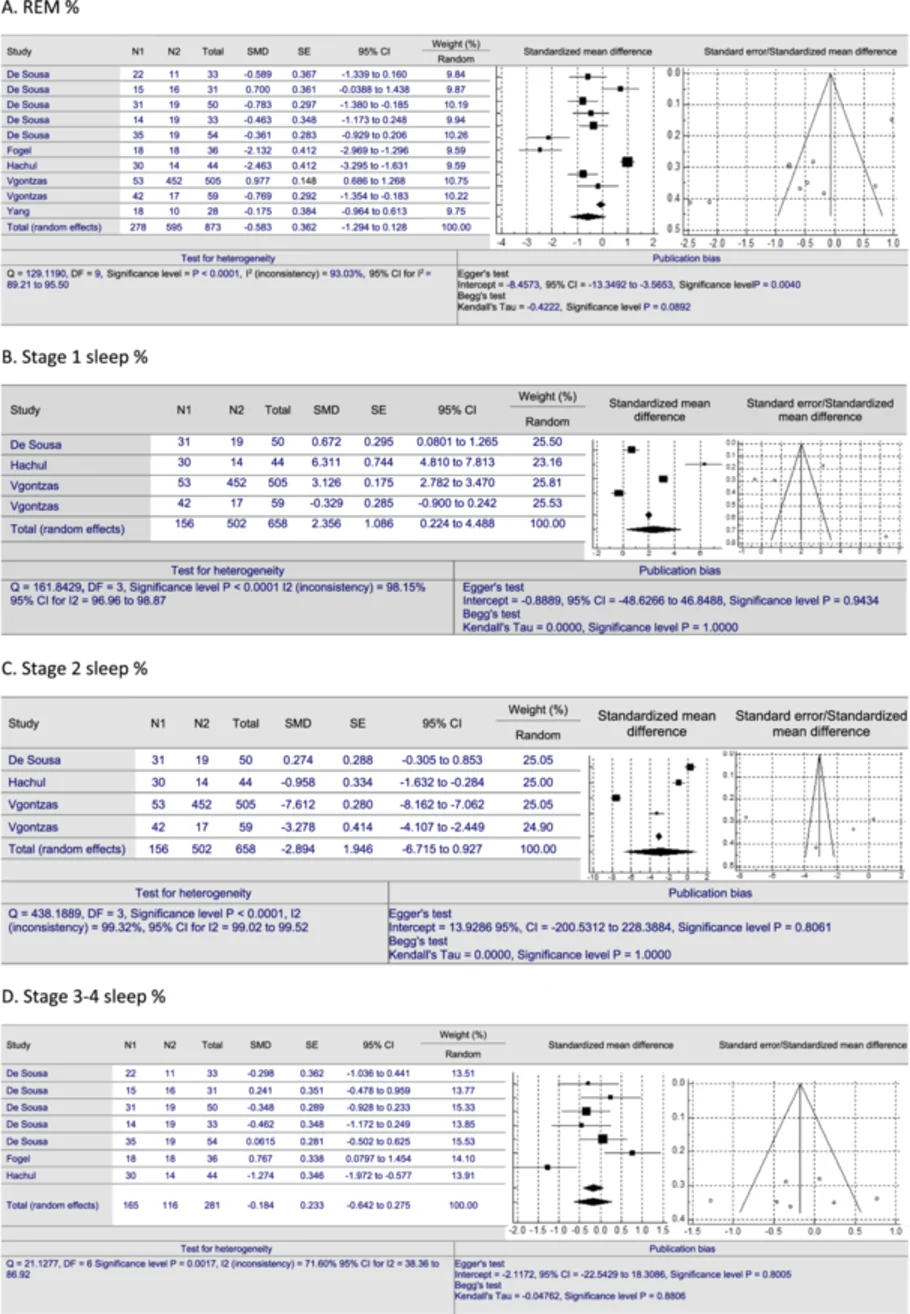
Forest plots of objective sleep architecture measures comparing women with polycystic ovary syndrome (PCOS) and controls. Effect sizes are presented as standardized mean differences (SMDs) with 95% confidence intervals (CIs). Funnel plots show the assessment of publication bias. Panels represent REM percentage (A), Stage 1 sleep percentage (B), Stage 2 sleep percentage (C), and stages 3‐4 sleep percentage (D).

The analysis of REM percentage in PCOS patients compared to the control group included ten studies with a total of 873 participants (278 in the PCOS group and 595 in the control group). The random effects model yielded an effect size of −0.583 (95% CI: −1.294 to 0.128, *p *= 0.11), indicating no significant difference (Figure 3).

The heterogeneity analysis showed high heterogeneity among the included studies (I^2^ = 93.03%, *p *< 0.001). The publication bias tests indicated potential bias (Egger's test *p *= 0.004, Begg's test *p *= 0.09) (Figure 3).

#### 3.5.2 Stage 1

The analysis of stage 1 percentage in PCOS patients compared to the control group included four studies with a total of 658 participants (156 in the PCOS group and 502 in the control group). The random effects model yielded an effect size of 2.356 (95% CI: 0.224 to 4.488, *p *= 0.03), indicating a significant difference between the two groups (Figure 3).

The heterogeneity analysis showed high heterogeneity among the included studies (I^2^ = 98.15%, *p *< 0.001). The publication bias tests (Egger's and Begg's) did not show significant bias (Figure 3).

#### 3.5.3 Stage 2

The analysis of stage 2 percentage included four studies with a total of 658 participants (156 in the PCOS group and 502 in the control group). The random effects model showed an effect size of −2.894 (95% CI: −6.715 to 0.927, *p *= 0.14), which was not statistically significant (Figure 3).

The heterogeneity analysis revealed very high variability among the included studies (I^2^ = 99.32%, *p*< 0.001). The publication bias tests (Egger's and Begg's) did not indicate significant bias (Figure 3).

#### 3.5.4 Stage 3 and 4

The analysis of stage 3 and 4 percentages in PCOS patients versus the control group included seven studies with a total of 281 participants (165 in the PCOS group and 116 in the control group). The random effects model indicated no significant difference between groups (effect size: −0.184, 95% CI: −0.642 to 0.275, *p* = 0.43) (Figure 3). Moderate heterogeneity was observed (I^2^ = 71.60%, *p* = 0.002), but no significant publication bias was detected (Figure 3).

### 3.6 Total Sleep Time

**Figure 4:**
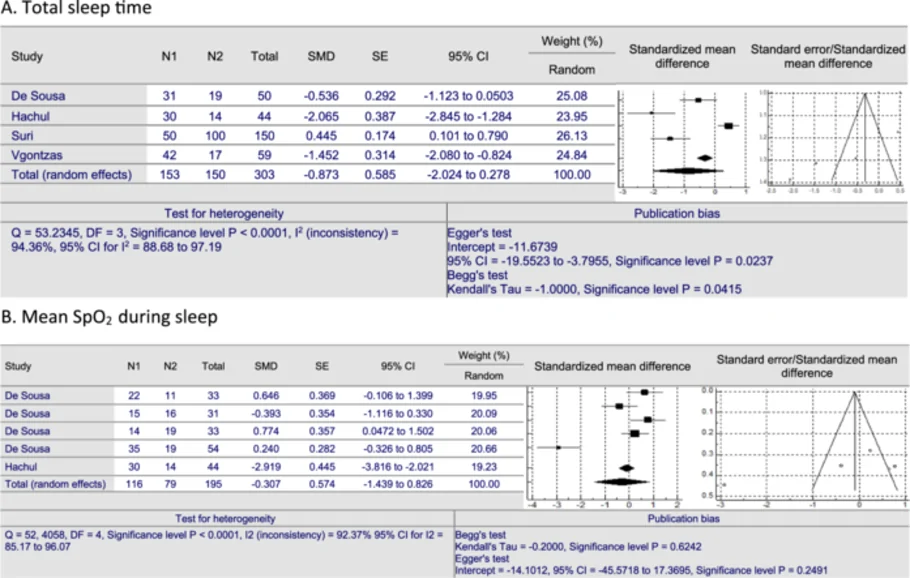
Forest plots of total sleep time (A) and mean oxygen saturation (B) comparing women with polycystic ovary syndrome (PCOS) and controls. Effect sizes are presented as standardized mean differences (SMDs) with 95% confidence intervals (CIs). Funnel plots show the assessment of publication bias.

The analysis of total sleep time in PCOS patients compared to the control group included four studies with a total of 303 participants (153 in the PCOS group and 150 in the control group). The random effects model indicated non‐significant difference between the groups (effect size: −0.873, 95% CI: −2.024 to 0.278, *p* = 0.14) (Figure 4). High heterogeneity was observed (I^2^ = 94.36%, *p* < 0.001), and potential publication bias was detected (Egger's test *p* = 0.024, Begg's test *p* = 0.04) (Figure 4).

### 3.7 Mean O2 Saturation

The analysis of mean O2 saturation during sleep included five studies with a total of 195 participants (116 in the PCOS group and 79 in the control group). The random effects model indicated no significant difference between groups (effect size: −0.307, 95% CI: −1.439 to 0.826, *p* = 0.59) (Figure 4).

High heterogeneity was observed (I^2^ = 92.37%, *p* < 0.001), but no significant publication bias was detected (Figure 4).

### 3.8 Subjective Measures

#### 3.8.1 PSQI

**Figure 5:**
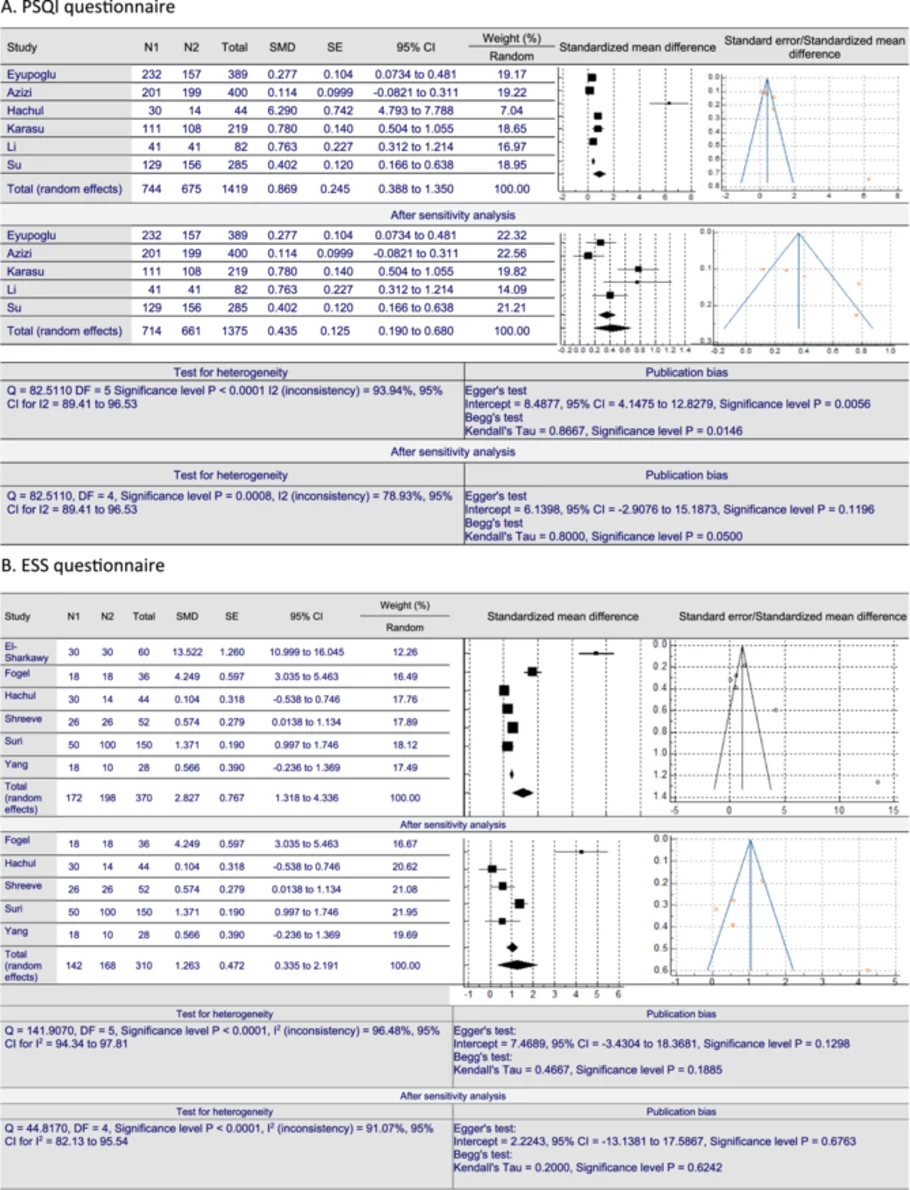
Forest plots of the Pittsburgh Sleep Quality Index (PSQI) (A) and Epworth Sleepiness Scale (ESS) (B) scores comparing women with polycystic ovary syndrome (PCOS) and controls. Effect sizes are presented as standardized mean differences (SMDs) with 95% confidence intervals (CIs). Funnel plots show the assessment of publication bias.

The analysis of the PSQI questionnaire before sensitivity analysis included six studies with a total of 1,419 participants (744 in the PCOS group and 675 in the control group). The random effects model yielded an effect size of 0.869 (95% CI: 0.388 to 1.350, *p* < 0.001), supporting the presence of a significant difference between groups (Figure 5).

The heterogeneity analysis showed high heterogeneity among the included studies (I^2^ = 93.94%, *p* < 0.001). The publication bias tests indicated potential bias (Egger's test *p* = 0.006, Begg's test *p* = 0.015) (Figure 5).

After sensitivity analysis, with the removal of the Hachul study, the analysis included five studies with a total of 1,375 participants (714 in the experimental group and 661 in the control group). The random effects model yielded a smaller effect size of 0.435 (95% CI: 0.190 to 0.680, *p* = 0.001), indicating a significant difference between the two groups (Figure 5).

The heterogeneity analysis showed reduced heterogeneity among the included studies after sensitivity analysis (I^2^ = 78.93%, *p* < 0.001). The publication bias tests (Egger's and Begg's) did not show significant bias after sensitivity analysis (Figure 5). The sensitivity analysis confirmed the robustness of the findings, with reduced heterogeneity and no significant publication bias.

#### 3.8.2 ESS

The analysis of the ESS questionnaire before sensitivity analysis included six studies with a total of 370 participants (172 in the PCOS group and 198 in the control group). The random effects model yielded an effect size of 2.827 (95% CI: 1.318 to 4.336, *p* < 0.001), indicating a significant difference between the two groups (Figure 5). The heterogeneity analysis showed high heterogeneity among the included studies (I^2^ = 96.48%, *p* < 0.001). The publication bias tests (Egger's and Begg's) did not show significant bias before sensitivity analysis (Figure 5).

After sensitivity analysis, the analysis included five studies with a total of 310 participants (142 in the PCOS group and 168 in the control group. The random effects model yielded an effect size of 1.263 (95% CI: 0.335 to 2.191, *p* = 0.008), still supporting the presence of a significant difference between the two groups (Figure 5).

The heterogeneity analysis showed reduced heterogeneity among the included studies after sensitivity analysis (I^2^ = 91.07%, *p* < 0.001). The publication bias tests did not show significant bias after sensitivity analysis (Figure 5). The sensitivity analysis confirmed the robustness of the findings, with reduced heterogeneity and no significant publication bias.

While a formal statistical subgroup meta‐analysis was not feasible due to the lack of data in the primary studies, we qualitatively summarized the phenotypic observations as follows:

The available evidence suggests that obesity explains part but not all of the observed sleep abnormalities. De Sousa et al. reported lower sleep efficiency (76.6%–77.1% vs. 92.3%), prolonged sleep‐onset latency (26.1–35.9 vs. 9.7–14.2 min), and lower REM sleep (8.8%–9.2% vs. 12.0%–13.6%) in obese women with PCOS compared with normal‐weight controls, whereas these differences were generally attenuated when obese controls were used [26, 29]. In the study by De Sousa et al. [29], obese women with PCOS with and without metabolic syndrome showed comparable apnea‐hypopnea index (0.73 vs. 0.82 events/h), sleep efficiency (77.1% vs. 73.1%), REM sleep (9.2% vs. 7.8%), and sleep‐onset latency (35.9 vs. 29.3 min), with no significant differences between two PCOS subgroups. Similarly, Hachul et al. found no significant differences between hyperandrogenic and non‐hyperandrogenic PCOS in PSQI score (8.8 vs. 8.2), ESS score (8.2 vs. 8.8), apnea‐hypopnea index (12.8 vs. 6.1 events/h; *p* = 0.507), or other polysomnographic measures after adjustment for age [32]. In contrast, Vgontzas et al. reported that the association between PCOS and SDB remained significant after adjustment for BMI. SDB requiring treatment was present in 8.3% versus 0% of non‐obese women with PCOS and controls and in 19.5% versus 4.5% of obese women (OR, 5.1; 95% CI, 1.1–31.3; *p* = 0.03). Likewise, excessive daytime sleepiness remained more frequent in women with PCOS in both non‐obese (75.0% vs. 22.5%; OR, 10.3; 95% CI, 2.5–60.0; *p* = 0.0005) and obese (82.1% vs. 54.5%; OR, 3.8; 95% CI, 1.4–11.6; *p* = 0.007) subgroups [45]. Another study by Vgontzas et al. found that women with PCOS had lower stage 2 sleep and higher slow‐wave sleep than obese controls, lower REM sleep and longer REM latency than normal‐weight controls [46].

### 3.9 Certainty of Evidence Assessment

The certainty of evidence for all objective and subjective outcomes was consistently rated as very low. Full details of the GRADE assessment, including the rationale for all downgrades, are presented in Table 3.

**Table 3:** Certainty of evidence assessment using GRADE.

Outcome	No. of studies (participants)	Effect estimate (SMD [95% CI])	Final certainty (GRADE)	Rationale for rating (downgrading factors)
Objective measures (polysomnography)				
Apnea index	5 (*N* = 201)	0.126 [−0.16 to 0.41]	⊕◯◯◯ very low	Downgraded 4 levels from low: risk of bias (↓ 2): serious confounding issues; imprecision (↓ 1): CI crosses null; publication bias (↓ 1): Egger's test *p* = 0.008.
Hypopnea index	5 (*N* = 201)	0.231 [−0.05 to 0.51]	⊕◯◯◯ very low	Downgraded 3 levels from low: risk of bias (↓ 2): serious confounding issues; imprecision (↓ 1): CI crosses null.
Apnea‐hypopnea index (AHI)	10 (*N* = 424)	1.030 [0.41 to 1.65]	⊕◯◯◯ very low	Downgraded 5 levels from low: risk of bias (↓ 2): serious confounding issues in ≈33% of studies (NOS Comparability ⋆); inconsistency (↓ 2): very high heterogeneity (I^2^ = 88%); publication bias (↓ 1): Egger's test *p* = 0.015.
Arousal index	3 (*N* = 128)	1.581 [−0.41 to 3.57]	⊕◯◯◯ very low	Downgraded 5 levels from low: risk of bias (↓ 2): serious confounding issues; inconsistency (↓ 2): very high heterogeneity (I^2^ = 95%); imprecision (↓ 1): CI crosses null widely.
Stage 1 sleep %	4 (*N* = 658)	2.356 [0.22 to 4.49]	⊕◯◯◯ very low	Downgraded 5 levels from low: risk of bias (↓ 2): serious confounding issues; inconsistency (↓ 2): extreme heterogeneity (I^2^ = 98%); imprecision (↓ 1): wide confidence interval.
Stage 2 sleep %	4 (*N* = 658)	−2.894 [−6.72 to 0.93]	⊕◯◯◯ very low	Downgraded 5 levels from low: risk of bias (↓ 2): serious confounding issues; inconsistency (↓ 2): extreme heterogeneity (I^2^ = 99%); imprecision (↓ 1): CI crosses null widely.
Stage 3/4 sleep %	7 (*N* = 281)	−0.184 [−0.64 to 0.27]	⊕◯◯◯ very low	Downgraded 4 levels from low: risk of bias (↓ 2): serious confounding issues; inconsistency (↓ 1): high heterogeneity (I^2^ = 72%); imprecision (↓ 1): CI crosses null.
REM %	10 (*N* = 873)	−0.583 [−1.29 to 0.13]	⊕◯◯◯ very low	Downgraded 6 levels from low: risk of bias (↓ 2): serious confounding issues; inconsistency (↓ 2): very high heterogeneity (I^2^ = 93%); imprecision (↓ 1): CI crosses null; publication bias (↓ 1): Egger's test *p* = 0.004.
Total sleep time	4 (*N* = 303)	−0.873 [−2.02 to 0.28]	⊕◯◯◯ very low	Downgraded 6 levels from low: risk of bias (↓ 2): serious confounding issues; inconsistency (↓ 2): very high heterogeneity (I^2^ = 94%); imprecision (↓ 1): CI crosses null; publication bias (↓ 1): Egger's test *p* = 0.024.
Mean O2 saturation	5 (*N* = 195)	−0.307 [−1.44 to 0.83]	⊕◯◯◯ very low	Downgraded 5 levels from low: risk of bias (↓ 2): serious confounding issues; inconsistency (↓ 2): very high heterogeneity (I^2^ = 92%); imprecision (↓ 1): CI crosses null widely.
Subjective measures				
PSQI score (post‐sensitivity)	5 (*N* = 1,375)	0.435 [0.19 to 0.68]	⊕◯◯◯ very low	Downgraded 3 levels from low: risk of bias (↓ 2): serious confounding issues; inconsistency (↓ 1): high heterogeneity (I^2^ = 79%).
ESS score (post‐sensitivity)	5 (*N* = 310)	1.263 [0.33 to 2.19]	⊕◯◯◯ very low	Downgraded 4 levels from low: risk of bias (↓ 2): serious confounding issues; inconsistency (↓ 2): very high heterogeneity (I^2^ = 91%).

## 4 Discussion

This meta‐analysis of 21 studies investigated sleep disturbances in patients with PCOS. Various parameters were analyzed, including objective and subjective measures. In terms of objective measurements, the findings revealed significant difference in certain SDB index, namely apnea‐hypopnea index, between women with PCOS and the control group. Other findings demonstrated differences in sleep architecture, including longer stage 1 in the PCOS group. However, REM sleep, stage 2, and stages 3 and 4 percentages showed no significant differences between groups. Furthermore, when it comes to the subjective measures, the study highlighted a significant association between sleep quality and PCOS. Women with PCOS exhibited significantly higher scores on both PSQI and ESS questionnaires compared to the non‐PCOS control group.

No significant association was found with either apnea or hypopnea and arousal indices. This is consistent with the meta‐analysis conducted by He et al., which reported higher prevalence of obstructive sleep apnea‐hypopnea syndrome among women with PCOS [47]. In both studies, the included population was women with PCOS and obesity, making definitive conclusions challenging. The analyzed population in the He et al. study mainly consisted of Caucasians, which elevates the risk of selection bias; in this study, diverse studies from various countries and ethnicities were included, which may improve the generalizability of the findings. Similarly, another meta‐analysis by Kahal et al. reported a higher prevalence of OSA among women with PCOS versus the control group [16]. This study included 17 studies from 8 electronic databases. A high degree of heterogeneity was observed across the included studies. A similar heterogeneity was also evident in the included studies of this review, as in the diagnosis of OSA. In a large population‐based cohort study involving 76978 women with PCOS and 143077 controls in the United Kingdom, a higher incidence rate of OSA was reported for women with PCOS. No significant impact of obesity on the association between OSA and PCOS was found. No other variables relevant to sleep quality or breathing indices were reported in this study [48].

This study also demonstrated differences in sleep architecture. Women with PCOS had significantly longer stage 1 compared to the control groups. In contrast, no significant differences were observed in stage 2 sleep or stages 3 and 4. Likewise, REM sleep percentage did not differ significantly between groups. Evidence suggests that individuals with PCOS may spend more time in lighter sleep stages of 1 and 2 compared to healthy controls, potentially contributing to poorer sleep quality and daytime fatigue [20, 49]. A previous meta‐analysis by Wang et al. demonstrated that REM sleep was significantly higher in PCOS than in controls [50]. This variability may be attributed to the high heterogeneity and potential publication bias, necessitating further investigation into this area.

The findings of this study underscore the significant impact of PCOS on sleep quality and daytime sleepiness, as measured by PSQI and ESS. PSQI is a widely used questionnaire to evaluate sleep quality by assessing subjective sleep quality, sleep duration, sleep latency, habitual sleep efficiency, sleep disturbances, daytime dysfunction, and use of sleep medication [51]. The analysis of the PSQI score revealed a significant difference in sleep quality between women with PCOS and the control group. Also, in this study, ESS analysis revealed significant differences between the PCOS and control groups. ESS measures daytime sleepiness [52]. Excessive daytime sleepiness is a common complaint among PCOS patients. These results align with previous evidence suggesting that sleep disturbances are an under‐recognized comorbidity in women with PCOS. Moran et al. systematically reviewed sleep disturbances in women with PCOS. They found that women with PCOS had poorer sleep quality and higher daytime sleepiness compared to controls. They highlighted the roles of OSA, obesity, and insulin resistance in contributing to sleep disturbances [49].

Other sleep‐related questionnaires and tools have been used in PCOS patients. The BQ is used to screen for OSA risk. It helps identify patients who may need further evaluation with polysomnography [53]. Kangwolkij et al. found that women with PCOS were more likely to have a high risk for OSA based on BQ compared to age‐ and BMI‐matched controls. This indicated that PCOS may independently contribute to increased risk of OSA [54]. These findings were compatible with three previous studies assessing the risk of OSA based on BQ [55, 56, 57]. The AIS is a validated tool used to measure insomnia based on ICD‐10. It includes eight items assessing sleep induction, nighttime awakening, early morning awakening, sleep duration, sleep quality, and daytime function [58]. Existing studies using AIS in PCOS populations have not reported significant findings regarding insomnia prevalence or severity [59, 60].

The available evidence suggests that obesity explains part, but not all, of the observed sleep abnormalities. De Sousa et al. reported poorer sleep efficiency, prolonged sleep‐onset latency, and lower REM sleep in obese women with PCOS compared with normal‐weight controls, whereas differences were generally attenuated when obese controls were used [26, 29]. Neither metabolic syndrome nor hyperandrogenism appeared to substantially influence sleep parameters within the PCOS group [29, 32]. In contrast, Vgontzas et al. found that the association of PCOS with SDB and excessive daytime sleepiness remained significant after adjustment for BMI, suggesting that factors beyond obesity may also contribute to sleep disturbances in PCOS [45]. Another study by the same group also identified differences in sleep architecture and inflammatory markers between women with PCOS and control groups, supporting the contribution of both obesity‐related and PCOS‐specific mechanisms [46]. Several biological mechanisms could underlie the link between PCOS and sleep disturbances. Hyperandrogenism, insulin resistance, obesity, and chronic low‐grade inflammation are all central to PCOS, and each has been linked to SDB and poorer sleep quality [13, 61]. Insulin resistance can fuel systemic inflammation and obesity, both of which can increase the risk of OSA [61]. Hormonal and metabolic disturbances may also reshape sleep architecture, and obesity in particular tends to worsen upper airway collapse and nighttime drops in oxygen [9, 13, 62, 63]. Circadian disruption may matter too: a multicenter study linked night‐shift work to PCOS and found altered circadian rhythmicity and circadian rhythm disruption in ovarian granulosa cells, hinting at a connection between disrupted body clocks, metabolic problems, and sleep [64]. Still, we need more longitudinal and mechanistic studies before any of this can be said with confidence.

It is important to acknowledge that the clinical heterogeneity observed in this meta‐analysis may have influenced the pooled estimates. Populations varied in age, BMI or obesity status, the applied PCOS diagnostic criteria, and the assessment approach for sleep disturbances, ranging from polysomnography to validated subjective questionnaires. Such differences likely shaped the size of the associations we observed and may account, at least in part, for the wide between‐study variability seen across several of the meta‐analyses. Furthermore, the certainty of evidence for most outcomes was generally rated as very low according to the GRADE approach, mainly because of risk of bias, inconsistency, imprecision, and potential publication bias. These discrepancies suggest that while our pooled estimates provide a robust overview, the findings should be interpreted with caution.

Our findings have significant implications for the clinical management of women with PCOS. Beyond the traditional focus on hormonal and reproductive health, our results suggest sleep disturbances should be integrated into the routine clinical assessment of these patients. Given the complex interplay between sleep and PCOS, a more proactive screening approach is warranted. Specifically, clinicians should consider implementing formal, standardized tools for evaluating sleep quality and related disturbances in patients. Such an approach aligns with the necessity for a multidisciplinary management model, where endocrinologists, gynecologists, and potentially sleep specialists collaborate to provide holistic care. Moving toward this integrated model could facilitate early identification of complications and allow for more personalized, comprehensive therapeutic interventions aimed at improving long‐term health outcomes in women with PCOS.

### 4.1 Strengths and Limitations of This Study

This study has several strengths. A comprehensive and systematic literature search was performed in international databases of PubMed/MEDLINE, Web of Science, and Scopus from database inception through August 2024. Both objective (polysomnography) and subjective (validated sleep questionnaires) measures of sleep were included, providing a multidimensional assessment of sleep disturbances. Certainty of evidence was assessed using GRADE. Furthermore, studies from multiple countries, encompassing diverse populations, were analyzed, thereby enhancing the generalizability of the findings.

Several limitations should be acknowledged. Some relevant studies could not be included due to a lack of full‐text availability, which may introduce selection bias. Additionally, the available evidence is largely observational and of limited methodological strength. The principal gap that remains is prospective research capable of clarifying the temporal sequence of this relationship and determining whether systematic screening for and treatment of sleep disorders leads to improved outcomes in this population.

## 5 Conclusion

This study provides evidence for the strong association between PCOS and sleep disturbances. The findings highlight significant differences in some SDB indices, sleep structure, and objective sleep quality measures between women with PCOS and control groups. Subjective sleep measures showed more pronounced differences than some objective assessments, suggesting a greater impact on perceived sleep disturbances. However, because the available evidence is based primarily on observational studies with generally very low certainty, these findings should be interpreted as associations rather than causal relationships. Well‐designed prospective and longitudinal studies are needed to clarify the temporal and causal relationships between PCOS and sleep disturbances.

## Author Contributions

**Arefeh Tabashiri:** conceptualization, investigation, writing – original draft, methodology, writing – review and editing, data curation, visualization, project administration, software. **Pourya Kanani:** investigation, writing – original draft, methodology, data curation, writing – review and editing, visualization, project administration. **Marzieh Saei Ghare Naz:** writing – original draft, methodology, writing – review and editing, formal analysis, software, data curation, supervision. **Fahimeh Ramezani Tehrani:** conceptualization, investigation, writing – original draft, validation, methodology, project administration, supervision, resources.

## Funding

The authors have nothing to report.

## Conflicts of Interest

The authors declare no conflicts of interest.

## AI Disclosure

No AI tools were used at any stage of this manuscript's preparation, including study design, data analysis, or writing.

## Author Declaration

All authors have reviewed and approved the final version of the manuscript. FRT had full access to all data in this study and takes full responsibility for the integrity of the data and the accuracy of the data analysis.

## 1 Transparency Statement

Fahimeh Ramezani Tehrani confirms that this manuscript provides an honest, accurate, and transparent account of the reported study; that no significant aspects of the study have been left out; and that any deviations from the planned (and, where applicable, registered) study have been clarified.

## Supporting information


Supporting File 1

Supporting File 2

## Data Availability

The data that support the findings of this study are available on request from the corresponding author. The data are not publicly available due to privacy or ethical restrictions. The data that support the findings of this study are available from the corresponding author upon reasonable request. The corresponding author had full access to all of the data in this study and takes complete responsibility for the integrity of the data and the accuracy of the data analysis.
