# Supplementary material for: Polycystic Ovary Syndrome and Subjective and Objective Assessments of Sleep Quality: A Systematic Review and Meta‐Analysis
Source: Health Sci Rep. 2026 Sep 14;9(9):e73223. doi: 10.1002/hsr2.73223 (PMC13575339; doi:10.1002/hsr2.73223)
Supplement: Supplementary file 1 [file HSR2-9-e73223-s001.docx]

S1 Table. Outcome reported of included studies.

| **Author** | **Variable (Unit)** | **Case** | **Control** |
| --- | --- | --- | --- |
| **Azizi et al. (1)** | PSQI | 9.93 ± 3.58 | 9.52 ± 3.58 |
| **Kangwolkij et al. (2)** | HDL (mg/dl) | 54.30 ± 14.70 | 58.70 ± 14.30 |
|  | Waist/Hip | 0.84 ± 0.06 | 0.79 ± 0.06 |
| **De Sousa et al. (3)** | Apnea index (events/hr) | 0.97 ± 1.29 | 1.32 ± 1.26 |
|  | Apnea-Hypopnea index (events/hr) | 1.03 ± 1.30 | 1.06 ± 0.79 |
|  | Hypopnea index (events/hr) | 0.05 ± 0.12 | 0.04 ± 0.13 |
|  | REM (% of TST) | 8.80 ± 5.79 | 12.04 ± 4.34 |
|  | SPO2 (%) | 97.86 ± 1.32 | 96.55 ± 2.91 |
|  | Stage3-4 (% of TST) | 33.16 ± 12.03 | 37.26 ± 16.01 |
| **De Sousa et al. (4)** | Apnea index (events/hr) | 0.83 ± 0.52 | 1.06 ± 1.04 |
|  | Apnea-Hypopnea index (events/hr) | 0.89 ± 0.60 | 1.09 ± 1.05 |
|  | Hypopnea index (events/hr) | 0.05 ± 0.11 | 0.03 ± 0.07 |
|  | REM (% of TST) | 10.05 ± 5.36 | 6.96 ± 2.99 |
|  | SPO2 (%) | 98.00 ± 1.51 | 98.56 ± 1.26 |
|  | Stage3-4 (% of TST) | 33.14 ± 14.04 | 37.26 ± 16.01 |
| **De Sousa et al. (5)** | Apnea index (events/hr) | 0.92 ± 1.16 | 0.44 ± 0.50 |
|  | Apnea-Hypopnea index (events/hr) | 0.95 ± 1.17 | 0.45 ± 0.50 |
|  | Hypopnea index (events/hr) | 0.04 ± 0.12 | 0.01 ± 0.05 |
|  | Tot sleep (min) | 317.19 ± 82.24 | 355.71 ± 45.32 |
|  | REM (% of TST) | 7.24 ± 4.79 | 10.84 ± 4.05 |
|  | Stage1 (% of TST) | 1.84 ± 1.77 | 0.83 ± 0.78 |
|  | Stage2 (% of TST) | 37.55 ± 12.92 | 33.96 ± 12.85 |
|  | Stage3-4 (% of TST) | 32.60 ± 13.27 | 37.17 ± 12.38 |
|  | FBS (mg/dl) | 83.70 ± 6.80 | 86.60 ± 6.50 |
|  | Testosterone (nmol/l) | 2.20 ± 0.80 | 1.70 ± 0.80 |
| **De Sousa et al. (6)** | Apnea index (events/hr) | 0.64 ± 0.60 | 0.65 ± 1.04 |
|  | Apnea-Hypopnea index (events/hr) | 0.73 ± 0.98 | 0.47 ± 0.52 |
|  | Hypopnea index (events/hr) | 0.09 ± 0.32 | 0.03 ± 0.10 |
|  | REM (% of TST) | 9.16 ± 4.11 | 10.84 ± 4.05 |
|  | SPO2 (%) | 97.79 ± 1.53 | 96.16 ± 2.36 |
|  | Stage3-4 (% of TST) | 31.01 ± 14.21 | 36.88 ± 10.92 |
|  | D. BP (mmHg) | 79.00 ± 8.00 | 76.00 ± 9.00 |
|  | S. BP (mmHg) | 132.00 ± 8.00 | 116.00 ± 12.00 |
|  | TG (mg/dl) | 198.00 ± 75.00 | 123.00 ± 70.00 |
|  | HDL (mg/dl) | 39.00 ± 10.00 | 50.00 ± 14.00 |
| **De Sousa et al. (7)** | Apnea index (events/hr) | 0.76 ± 0.47 | 0.44 ± 0.50 |
|  | Apnea-Hypopnea index (events/hr) | 0.79 ± 0.98 | 0.45 ± 0.50 |
|  | Hypopnea index (events/hr) | 0.03 ± 0.09 | 0.01 ± 0.05 |
|  | SPO2 (%) | 97.17 ± 1.47 | 96.79± 1.72 |
|  | REM (% of TST) | 9.34 ± 4.11 | 10.84 ± 4.05 |
|  | Stage3-4 (% of TST) | 37.96 ± 12.82 | 37.17 ± 12.38 |
| **El-Sharkawy et al. (8)** | FBS (mg/dl) | 125.23 ± 2.61 | 86.5 ± 1.65 |
| **Hachul et al. (9)** | Apnea-Hypopnea index (events/hr) | 9.2 ± 2.6 | 1.6 ± 3.2 |
|  | Arousal index (events/hr) | 18.9 ± 2.2 | 12.6 ± 3.2 |
|  | ESS | 8.5 ± 0.8 | 8.4 ± 1.2 |
|  | PSQI | 8.5 ± 0.5 | 4.6 ± 0.8 |
|  | SPO2 (%) | 95.9 ± 0.2 | 96.6 ± 0.3 |
|  | Tot Sleep (min) | 339.4 ± 11.2 | 366.8 ± 16.4 |
|  | REM (% of TST) | 17.3 ± 1.1 | 20.5 ± 1.6 |
|  | Stage1 (% of TST) | 11.5 ± 1.1 | 3.3 ± 1.6 |
|  | Stage2 (% of TST) | 45.9 ± 1.6 | 47.7 ± 2.3 |
|  | Stage3-4 (% of TST) | 25.2 ± 1.8 | 27.9 ± 2.6 |
| **Karasu et al. (10)** | PSQI | 7 ± 4.5 | 4 ± 3 |
| **Suri et al.(11)** | ESS | 12.5 ± 3.2 | 9.32 ± 1.7 |
|  | Tot sleep (min) | 402 ± 67.9 | 374.6 ± 57.6 |
| **Yang et al. (12)** | Waist/hip | 0.90 ± 0.01 | 0.82 ± 0.01 |
|  | Apnea-Hypopnea index (events/hr) | 0.79 ± 0.21 | 0.29 ± 0.09 |
|  | Arousal index (events/hr) | 11.20 ± 0.96 | 8.31 ± 1.30 |
|  | D. BP (mmHg) | 68.3 ± 2.14 | 71.1 ± 3.65 |
|  | S. BP (mmHg) | 109 ± 2.2 | 109 ± 4.14 |
|  | ESS | 9.63 ± 0.95 | 9 ± 1.29 |
|  | FBS (mg/dl) | 87.1 ± 1.38 | 89.3 ± 1.34 |
|  | REM (% of TST) | 23.3 ± 1.01 | 23.5 ± 1.27 |
|  | Testosterone (ng/ml) | 0.77 ± 0.10 | 0.41 ± 0.03 |
| **Li et al. (13)** | Testosterone (ng/ml) | 1.40 ± 0.51 | 0.91 ± 0.44 |
| **Eyupoglu et al. (14)** | PSQI | 6.20 ± 2.60 | 5.50 ± 2.40 |
| **Fogel et al. (15)** | Waist/hip | 0.88 ± 0.02 | 0.82 ± 0.01 |
|  | Apnea-Hypopnea index (events/hr) | 22.50 ± 6 | 6.70 ± 1.40 |
|  | ESS | 9.50 ± 0.90 | 5.80 ± 0.80 |
|  | REM (% of TST) | 11.80 ± 1.30 | 15.10 ± 1.70 |
|  | Stage3-4 (% of TST) | 22.50 ± 2.40 | 20.90 ± 1.60 |
|  | Testosterone (ng/dl) | 94.4 ± 8.5 | 22.8 ± 2.5 |
| **Li et al. (16)** | PSQI | 7.19 ± 3.10 | 4.93 ± 2.76 |
|  | TG (mmol/l) | 1.60 ± 1.04 | 0.94 ± 0.35 |
|  | Testosterone (nmol/l) | 2.89 ± 1.14 | 1.71 ± 0.33 |
| **Shreeve et al. (17)** | ESS | 7.96 ± 4.57 | 5.56 ± 3.61 |
| **Vgontzas et al. (18)** | REM (% of TST) | 18.7 ± 1.5 | 18.1 ± 0.4 |
|  | Stage1 (% of TST) | 5.3 ± 0.8 | 4.3 ± 0.2 |
|  | Stage2 (% of TST) | 66.3 ± 1.3 | 70.6 ± 0.4 |
| **Vgontzas et al. (19)** | Apnea-Hypopnea index (events/hr) | 1.20 ± 0.60 | 0.90 ± 0.40 |
|  | REM (% of TST) | 16.90 ± 1.10 | 18.00 ± 2.00 |
|  | Stage1 (% of TST) | 5.70 ± 0.90 | 6.00 ± 0.90 |
|  | Stage2 (% of TST) | 64.80 ± 1.50 | 70.30 ± 2.00 |
|  | Tot sleep (min) | 376.90 ± 12.40 | 394.90 ± 11.80 |
|  | D. BP (mmHg) | 79.50 ± 170 | 77.90 ± 3.10 |
|  | S. BP (mmHg) | 124.20 ± 2.00 | 127.90 ± 3.20 |
| **Nandalike (20)** | Apnea-Hypopnea index (events/hr) | 6.10 ± 6.90 | 2.20 3.20 |
|  | Arousal index (events/hr) | 12.70 ± 7.30 | 13.80 ± 5.50 |
|  | D. BP (mmHg) | 69.80 ± 11.20 | 66.40 ± .40 |
|  | S. BP (mmHg) | 122.90 ± 13.60 | 117.10 ± 9.60 |
|  | FBS (mg/dl) | 96.60 ± 20.10 | 85.30 ± 13.50 |
|  | HDL (Fasting) (mgdl) | 43.00 ± 10.90 | 50.50 ± 13.80 |
|  | TG (Fasting) (mgdl) | 125.50 ± 73.10 | 91.40 ± 38.90 |
| **Su et al. (21)** | PSQI | 6.82 ± 2.61 | 5.74 ± 2.74 |

PSQI: Pittsburgh Sleep Quality Index; HDL: High-Density Lipoprotein; Waist/Hip: Waist-to-Hip Ratio; REM: Rapid Eye Movement sleep; SPO2: Peripheral Oxygen Saturation; FBS: Fasting Blood Sugar; D. BP: Diastolic Blood Pressure; S. BP: Systolic Blood Pressure; TG: Triglycerides; ESS: Epworth Sleepiness Scale.

1.	Azizi Kutenaee M, Amirjani S, Asemi Z, Taghavi SA, Allan H, Kamalnadian SN, et al. The impact of depression, self-esteem, and body image on sleep quality in patients with PCOS: a cross-sectional study. Sleep Breathing. 2020;24(3):1027-34.

2.	Kangwolkij N, Sophonsritsuk A, Tantrakul V, Charakorn C, Tantanavipas S. Prevalence and Predictive Factors for Being High-risk of Obstructive Sleep Apnea Using Berlin Questionnaire in Polycystic Ovary Syndrome: Age - and BMI-matched study. Thai J Obstet Gynaecol. 2022;30(6):413-22.

3.	De Sousa G, Schlüter B, Buschatz D, Menke T, Trowitzsch E, Andler W, et al. A comparison of polysomnographic variables between obese adolescents with polycystic ovarian syndrome and healthy, normal-weight and obese adolescents. Sleep Breathing. 2010;14(1):33-8.

4.	De Sousa G, Schlüter B, Menke T, Trowitzsch E, Andler W, Reinehr T. Longitudinal analyses of polysomnographic variables, serum androgens, and parameters of glucose metabolism in obese adolescents with polycystic ovarian syndrome. Sleep Breathing. 2012;16(4):1139-46.

5.	de Sousa G, Schlüter B, Menke T, Trowitzsch E, Andler W, Reinehr T. Relationships between polysomnographic variables, parameters of glucose metabolism, and serum androgens in obese adolescents with polycystic ovarian syndrome. J Sleep Res. 2011;20(3):472-8.

6.	De Sousa G, Schlüter B, Menke T, Trowitzsch E, Andler W, Reinehr T. A comparison of polysomnographic variables between adolescents with polycystic ovarian syndrome with and without the metabolic syndrome. Metab Syndr Relat Disord. 2011;9(3):191-6.

7.	De Sousa G, Schlüter B, Buschatz D, Menke T, Trowitzsch E, Andler W, et al. The impact of insulin resistance and hyperandrogenemia on polysomnographic variables in obese adolescents with polycystic ovarian syndrome. Sleep Breathing. 2012;16(1):169-75.

8.	El-Sharkawy AA, Abdelmotaleb GS, Aly MK, Kabel AM. Effect of Metformin on Sleep Disorders in Adolescent Girls with Polycystic Ovarian Syndrome. J Pediatr Adolesc Gynecol. 2014;27(6):347-52.

9.	Hachul H, Polesel DN, Tock L, Carneiro G, Pereira AZ, Zanella MT, et al. Sleep disorders in polycystic ovary syndrome: influence of obesity and hyperandrogenism. Rev Assoc Med Bras (1992). 2019;65(3):375-83.

10.	Karasu AFG, Şahbaz CD, Doğu ZFE, Takmaz T, Çalı H, Tanoğlu B. Chronotype and sleep quality assessment of patients with polycystic ovary syndrome. Haseki Tip Bulteni. 2021;59(1):53-7.

11.	Suri J, Suri JC, Chatterjee B, Mittal P, Adhikari T. Obesity may be the common pathway for sleep-disordered breathing in women with polycystic ovary syndrome. Sleep Med. 2016;24:32-9.

12.	Yang H-P, Kang J-H, Su H-Y, Tzeng C-R, Liu W-M, Huang S-Y. Apnea–hypopnea index in nonobese women with polycystic ovary syndrome. International Journal of Gynecology & Obstetrics. 2009;105(3):226-9.

13.	Li H, Liu M, Zhang C. Women with polycystic ovary syndrome (PCOS) have reduced melatonin concentrations in their follicles and have mild sleep disturbances. BMC Women's Health. 2022;22(1).

14.	Eyupoglu ND, Aksun S, Ozturk M, Yildiz BO. Impact of social isolation during COVID-19 pandemic on health behaviors and weight management in women with polycystic ovary syndrome. Eating Weight Disord. 2022;27(7):2407-13.

15.	Fogel RB, Malhotra A, Pillar G, Pittman SD, Dunaif A, White DP. Increased prevalence of Obstructive Sleep Apnea syndrome in obese women with Polycystic Ovary Syndrome. J Clin Endocrinol Metab. 2001;86(3):1175-80.

16.	Li G, Hu J, Zhang S, Fan W, Wen L, Wang G, et al. Changes in Resting-State Cerebral Activity in Women With Polycystic Ovary Syndrome: A Functional MR Imaging Study. Front Endocrinol. 2020;11.

17.	Shreeve N, Cagampang F, Sadek K, Tolhurst M, Houldey A, Hill CM, et al. Poor sleep in PCOS; is melatonin the culprit? Hum Reprod. 2013;28(5):1348-53.

18.	Vgontzas AN, Legro RS, Bixler EO, Grayev A, Kales A, Chrousos GP. Polycystic ovary syndrome is associated with obstructive sleep apnea and daytime sleepiness: Role of insulin resistance. J Clin Endocrinol Metab. 2001;86(2):517-20.

19.	Vgontzas AN, Trakada G, Bixler EO, Lin HM, Pejovic S, Zoumakis E, et al. Plasma interleukin 6 levels are elevated in polycystic ovary syndrome independently of obesity or sleep apnea. Metab Clin Exp. 2006;55(8):1076-82.

20.	Nandalike K, Agarwal C, Strauss T, Coupey SM, Isasi CR, Sin S, et al. Sleep and cardiometabolic function in obese adolescent girls with polycystic ovary syndrome. Sleep Med. 2012;13(10):1307-12.

21.	Su N, Du C, Zhang Y, Deng L, Tang T, Zhuang B, et al. Retrospective investigation and analysis of sleep disorders on occurrence of polycystic ovary syndrome. Biomed Res. 2017;28(2):583-7.
