# Supplementary material for: Polycystic Ovary Syndrome and Subjective and Objective Assessments of Sleep Quality: A Systematic Review and Meta‐Analysis
Source: Health Sci Rep. 2026 Sep 14;9(9):e73223. doi: 10.1002/hsr2.73223 (PMC13575339; doi:10.1002/hsr2.73223)
Supplement: Supplementary file 2 [file HSR2-9-e73223-s002.docx]

The searched keywords included ("Polycystic Ovary Syndrome" OR “Syndrome, Polycystic Ovary” OR “Ovary Syndrome, Polycystic” OR “Polycystic Ovarian Syndrome” OR “Ovarian Syndrome, Polycystic” OR “Polycystic Ovary Syndrome 1” OR “Polycystic Ovaries” OR “PCOS” OR “PCO” OR “PCO1” OR “Bilateral PCOS” OR “Stein-Leventhal” OR “Stein Leventhal” OR “Stein-Leventhal Syndrome” OR “Syndrome, Stein-Leventhal” OR “Polycystic Ovary Disease” OR “Polycystic Ovarian Disease” OR “PCOD” OR “Sclerocystic Ovarian Degeneration” OR “Ovarian Degeneration, Sclerocystic” OR “Sclerocystic Ovary Syndrome” OR “Sclerocystic Ovarian Disease” OR “Sclerocystic Ovaries” OR “Sclerocystic Ovary” OR “Ovary, Sclerocystic” OR “Hyperandrogenism” OR “Androgens” OR “Anovulation” OR “Hirsutism”) AND (“Sleep Wake Disorders” OR “Disorder, Sleep Wake” OR “Disorders, Sleep Wake” OR “Sleep Wake Disorder” OR “Wake Disorder, Sleep” OR “Wake Disorders, Sleep” OR “Sleep Disorders” OR “Disorder, Sleep” OR “Disorders, Sleep” OR “Sleep Disorder” OR “Long Sleeper Syndrome” OR “Long Sleeper Syndromes” OR “Sleeper Syndrome, Long” OR “Sleeper Syndromes, Long” OR “Syndrome, Long Sleeper” OR “Syndromes, Long Sleeper” OR “Short Sleeper Syndrome” OR “Short Sleeper Syndromes” OR “Sleeper Syndrome, Short” OR “Sleeper Syndromes, Short” OR “Syndrome, Short Sleeper” OR “Syndromes, Short Sleeper” OR “Short Sleep Phenotype” OR “Phenotype, Short Sleep” OR “Phenotypes, Short Sleep” OR “Short Sleep Phenotypes” OR “Sleep Phenotypes, Short” OR “Sleep-Related Neurogenic Tachypnea” OR “Neurogenic Tachypnea, Sleep-Related” OR “Neurogenic Tachypneas, Sleep-Related” OR “Sleep Related Neurogenic Tachypnea” OR “Sleep-Related Neurogenic Tachypneas” OR “Tachypnea, Sleep-Related Neurogenic” OR “Tachypneas, Sleep-Related Neurogenic” OR “Subwakefullness Syndrome” OR “Subwakefullness Syndromes” OR “Syndromes, Subwakefullness” OR “Syndrome, Subwakefullness” OR “Sleep Apnea Syndromes” OR “Apnea Syndromes, Sleep” OR‎“Apnea Syndrome, Sleep” ‎ OR “Apnea, Sleep” OR “Sleep Apnea” OR “Apneas, Sleep” OR “Sleep Apneas” OR “Sleep Hypopnea” OR “Sleep Hypopneas” OR “Hypopnea, Sleep” OR “Hypopneas, Sleep” OR “Hypersomnia with Periodic Respiration” OR “Sleep Apnea, Mixed Central and Obstructive” OR “Mixed Central and Obstructive Sleep Apnea” OR “Sleep Apnea, Mixed” OR “Mixed Sleep Apnea” OR “Mixed Sleep Apneas” OR “Sleep Apneas, Mixed” OR “Sleep-Disordered Breathing” OR “Breathing, Sleep-Disordered” OR “Sleep Disordered Breathing”).
